# What Are Data?

**DOI:** 10.1177/10497323211015960

**Published:** 2021-05-24

**Authors:** Karin Olson

**Affiliations:** 1 University of Alberta, Edmonton, Alberta, Canada; 2 The University of British Columbia, Kelowna, British Columbia, Canada

In the rush to rearrange plans for data collection that fit with the new social distancing requirements of the Covid-19 pandemic, it is easy to lose track of what data are. I would like to invite readers to take a step back and think about this question. According to The Oxford Learner’s Dictionary (2021) data are “facts or information, especially when examined and used to find out things or to make decisions.”.

## “All Is Data”

Many years ago, Glaser (2001^1^, 2007) noted that “all is data.” Although originally written in reference to grounded theory, Glaser’s discussion of data has relevance for all qualitative researchers. He noted that data included not just what was said during an interview, for example, but also how it was told and the conditions under which it was reported. I learned about the importance of paying attention to this broader understanding of data in one of our early studies of fatigue in cancer patients when the nurses on an inpatient unit were helping us identify potential study participants (Olson et al., 2007). The nurses would say things like, “You should talk to Mr. X because he has significant fatigue.” When I asked them how they knew who had fatigue and who didn’t, they would just smile and say, “Just go talk to him.” Mr. X and others referred to us by the nurses who agreed to join the study described their fatigue in detail. We read these transcripts carefully and while coding, noted very short sentences of only 5 to 6 words. We went back and listened to the audio tapes again, and realized that the participants also spoke very slowly, pausing for long periods between sentences. These additional data about the rate of speech helped us understand the physical impact of fatigue on patients and how nurses recognized which patients were experiencing fatigue.

Data, as described by Glaser (2007), also include the non-verbal elements the researcher sees when they are able to be with participants face-to-face. Where does the participant look while they talk? How do they appear while they talk? What observations indicate emotions the participant might be feeling—a quivering chin, a small tear? These non-verbal data elements are missed when one collects data via email, chat, phone, or documents. The availability of non-verbal data helps the researcher see patterns in the data more clearly.

Modes of data collection are another factor that may influence the kind of data one is able to obtain, and hence its influence its interpretation. Roberts (2007) notes that while there are situations in which the use of more than one mode of data collection within a study is warranted, this practice makes it difficult to know whether differences among participants are due to the mode by which the data were collected or some other factors. There is also some evidence that the mode of data collection influences the data that participants provide. In a study comparing face-to-face focus groups and on-line focus groups in two different countries, Graffigna and colleagues (2008) found that the mode of data collection led to important differences in data that were shared, and that these differences were consistent across both countries studied.

Data are collected by researchers, and thus those data that are collected are limited by what the researcher apprehends. For this reason, one must remember that the data are incomplete. This is why it is important to remain in the field for prolonged periods, to interview participants more than once, and to use multiple data sources. Read and re-read data, and ask questions like: What else is happening here? What did I miss when I read this information before? What do I need to ask this participant when I interview them again? Careful notes must be kept during this process to ensure that the ideas that underpin interpretation of data are documented.

## Data Are Socially Constructed for a Purpose

Hubbard (1988) notes that the construction of data has become a “social enterprise” (p. 5). Taken together with the dictionary definition of data noted above, think about how the purpose for which data may be used informs its construction. Data may bear little resemblance to the researcher’s sense of “truth” or “reality” (Glaser, 2007). These data are, however, indicators of the realities of participants. This is why data, as described broadly by Glaser, are so important. As researchers carefully study data, they begin to see patterns in the data and understand how participants use data. When comparing patterns in data in subsequent interviews and across participants, the researcher can compare emerging ideas about the meanings within these data, gradually refining these insights based on new data, until arriving at meanings that require no further adjustment, even when new data are added.

Social construction is about how relationships and frames of reference give rise to the meanings of things that happen in daily life. “Through participation in relationships the world comes to be what it is for us” (Gergen, 2015, pp. 4–5). This seems like a simply notion, until one stops to consider that technology, for example, is playing an increasing role in the construction of data. Regardless of whether we study data from machines found in intensive care units or from personal fitness devices, this technology was made by another person to collect a specific type of information for a specific purpose. An explicit description of the relationships and frames of reference that have an influence on the construction of data show the context in which the data were collected and help to shape its meanings .

I have had to relearn the importance of the social construction of data many times. For example, in our study on depression, I was puzzled why we had not recruited any male participants (Porr et al., 2010). A male colleague suggested that perhaps the problem was that recruitment posters asked interested individuals to call me. We then recruited a male Research Assistant to assist with data collection, replacing my name with his on the recruitment posters—thereby changing the way data were collected. Within a short period, men called daily to express interest in the study. This experience speaks to deeply rooted societal values about how men should “be,” and about the stigma associated with mental illness, particularly among men. Without the insights of my male colleague, I would have missed this key point about the nature of mental illness completely.

The social construction of data means that it also includes the researcher’s reflections on the data. The danger here is that it is easy to drift so far into one’s own thoughts that the participant’s story gets lost. For this reason, researchers may find in useful to maintain a journal where they can track links between their own reflexive notes and the data shared by participants.

One way to learn more about how data are socially constructed is to examine how it is used. The revised model for developing complex interventions developed by the Medical Research Council (Craig et al., 2008) clearly shows how both quantitative and qualitative data can be used to develop and test complex interventions and evaluate their outcomes. On the other hand, the revised hierarchy of evidence pyramid published nearly 10 years later in the *British Medical Journal*, however, shows a clear preference for using only quantitative data as evidence (Murad et al., 2016). In fact, studies that use qualitative data are not included in this hierarchy of evidence pyramid. This realization triggers several questions: Why are some data not deemed useful? Who gets to decide what data are useful? Why can a whole body of evidence generated through qualitative research simply be ignored? What potential benefits are omitted when qualitative data are not included in the evidence-generation process?

Finally, it is worthwhile to ask whether all facts and pieces of information are data. Going back to the dictionary definition above, it is hard to imagine facts and information that simply exist but are not used for something. Gergen (2015) notes that while reality exists separate from data, reality only develops meaning when it is considered from the standpoint of relationship.

The take-home message here is to remember that data don’t just happen. Rather, data are constructed as part of a social enterprise for the purpose of understanding something or making decisions. Thinking about data broadly, as more than just text, provides the researcher with information about the realities of participants.

## Notes

1. “Although data is plural ‘is’ sounds better” (Glaser, 2001, p. 145).
